# Forestry Alters Foraging Efficiency and Crop Contents of Aphid-Tending Red Wood Ants, *Formica aquilonia*

**DOI:** 10.1371/journal.pone.0032817

**Published:** 2012-03-13

**Authors:** Therese Johansson, Heloise Gibb

**Affiliations:** Department of Wildlife, Fish, and Environmental Sciences, University of Agricultural Sciences, Umeå, Sweden; University of Arizona, United States of America

## Abstract

Forest management alters species behaviours, distributions and interactions. To evaluate forestry effects on ant foraging performance, we compared the quality and quantity of honeydew harvested by ants among clear-cuts, middle-aged and mature spruce-dominated stands in boreal forests in Sweden. Honeydew quality was examined using honeydew collected by squeezing the gasters of laden *Formica aquilonia* workers. We used fifteen laden individuals at each study site (four replicates of each stand age) and analysed honeydew chemical composition with gas chromatography-mass spectroscopy. To compare the quantity of honeydew collected by individual ants, we collected and weighed five ants moving up and five ants moving down each of ten trees at the twelve sites (totally 1200 ants). The concentration of trehalose in honeydew was lower in clear-cuts compared with middle aged and mature stands, and similar trends were shown for sucrose, raffinose and melezitose, indicating poorer honeydew quality on clear cuts. Concentrations of the amino acid serine were higher on clear-cuts. The same trend occurred for glutamine, suggesting that increased N-uptake by the trees after clear cutting is reflected in the honeydew of aphids. Ants in mature stands had larger heads and carried proportionally more honeydew and may therefore be more efficient foragers. Human alternation of habitats through clear-cutting thus affects food quality and worker condition in *F. aquilonia*. This is the first study to show that honeydew quality is affected by anthropogenic disturbances, likely contributing to the reduction in size and abundance of *F. aquilonia* workers and mounds after clear cutting.

## Introduction

Anthropogenic disturbance, mainly through urbanization, forestry and agriculture, has severe impacts on ecosystems worldwide, affecting ecosystem processes, habitat structure and species composition [1], [2], [3], [4], [5]. Effects on species interactions, such as competition, predation and parasitism are also commonly reported [6], [7], [8]. Although effects of anthropogenic disturbances on pollination are well known [9], [10], effects on other mutualisms, such as that commonly occurring between ants and hemiptera, are poorly understood [11].

Mutualisms with hemiptera are vital for ants in most ecosystems [12], [13]. The digestive systems of behaviourally and numerically dominant arboreal ants are highly specialised for the kind of carbohydrate-rich and protein-poor diets obtained from feeding on hemipteran exudates [14], [15]. Energetically expensive levels of activity and aggression may be promoted by the reliable sugar resource provided by the hemipterans [16], [17]. Because ant-hemiptera mutualisms result in significant quantities of energy being available to ants and therefore promote activities such as predation and seed dispersal that have broader effects on ecosystems, ant-hemiptera mutualisms may be considered to be keystone interactions [18].

Plant exudates play a key role for the carbon and nitrogen budgets of many ant species [19], [20]. Honeydew contains a broad range of sugars and amino acids of varying importance to ants. Previous studies suggest that honeydew quality varies between different environments [21], [22], [23]. Food quality may alter caste determination and size variation within castes [20], [24], with poor quality food resulting in smaller adult body size [25] and potentially altering foraging efficiency and size-dependent allocation of tasks [26]. Differences in food quality resulting from anthropogenic disturbances may therefore have significant effects on the success of ants.

Despite the keystone role of ant-hemiptera mutualisms, few studies have examined how they are affected by anthropogenic disturbances. In Scandinavia, intensive forestry has dramatically changed the structure and species composition of the boreal forest, with significant impacts on many species [27], [28], including the dominant red wood ants of the *Formica rufa* group [29], [30]. Land management alters ant use of hemipteran resources [11], [25], [31] and clear-cutting results in reduced colony survival, rates of reproduction and immunity for red wood ants [32], [33], [34]. Approximately 85% of the dry mass of the diet of red wood ants consists of honeydew from arboreal aphids [35], [36], yet it is unclear whether forest management affects the quality of this resource or the capacity of individual workers to harvest it. In this study, we address the following questions: 1) Does the composition of sugars and amino acids in ant-collected honeydew depend on stand age? and 2) Does worker body size have consistent effects on honeydew load in stands of different ages?

## Methods

### Study area and species

All study sites were situated in Norway spruce (*Picea abies*) dominated forest in the middle boreal zone [37] of northern Sweden between the latitudes of 63.6°N and 64.5°N and longitudes of 19.7°E and 20.7°E. Scots pine (*Pinus sylvestris*) and birches (*Betula pendula* and *B. pubescens*) were also common in the stands. The herb layer was dominated by dwarf shrubs (mostly *Vaccinium myrtillis*) and mosses (*Pleurozium schreberi*, *Hylocomium splendens*, *Sphangnum* spp.). On clear-cuts, *Deschampsia flexuosa* often dominated the herb layer. Soils were moist and of the sandy moraine type.

*Formica aquilonia* Yarrow is the most common *F. rufa* group species in the central boreal region of Fennoscandia [38]. In boreal forest, red wood ants (*Formica rufa* group) are ecologically dominant and form mutualisms with aphids [31], [35], [39]. In the study sites, *F. aquilonia* tends the aphids *Cinara pruinosa* (Hartig) and *C. piceicola* (Cholodkovsky)(*pers. obs.* by Heloise Gibb, identified by R. Danielsson, University of Lund). Previous studies report that *F. aquilonia* is affected by forest succession, being more common in old forests and in larger old-forest fragments [40].

We selected 12 study sites with established populations of the red wood ant *Formica aquilonia* for the study. Four study sites were in mature stands (tree age 80–100 years , mean basal diameter 7.4 cm, mean height 17.9 m), four in middle aged stands (30–40 years , mean basal diameter 4.9 cm, mean height 8.3 m) and four on clear cuts with 5–10 retention trees per ha (1–4 years, mean basal diameter 1.2 cm, mean height 2.1 m). Basal diameter includes all trees including saplings >1 cm. Stands of different ages were geographically interspersed. Each study plot supported several nests of *F. aquilonia*. All necessary permits were obtained for the described field studies.

### Honeydew collection

Honeydew was collected from fifteen laden *F. aquilonia* workers travelling down spruce trees after harvesting honeydew at each of the twelve study sites in July 2008. Ant crop contents have been shown to closely resemble the source, although there can be minor changes in sucrose, glucose and fructose concentrations due to enzymatic activity [12]. We assumed that any changes in sugar concentrations as a result of enzymatic activity would be consistent amongst stand ages because ants were of the same species and collected at similar distances from their nests. We did not directly control the source of the honeydew since we did not have access to the tree crowns while collecting honeydew. However, aphid surveys in the same stands were used to determine the main honeydew producers in the stands.

We collected honeydew from the crops of worker ants by gently squeezing their gasters, such that they regurgitated liquid into a 1.5 ml eppendorf vial. Cloudy samples representing contamination with haemolymph were discarded (as per Blüthgen et al. 2004) [21]. We weighed each vial before and after collecting the honeydew to obtain an exact weight for each sample. To prevent degradation, samples were kept on ice in the field, and then stored at −80°C on return to the laboratory.

### Chemical analyses

Samples weighing between 1 and 10 mg were analysed, resulting in 10–12 samples per stand and a total of 137 samples used in the analyses. The chemical composition (sugars and amino acids) of the honeydew were analysed with gas chromatography-mass spectroscopy (GC-MS) at the Metabolomics facilities at Umeå University.

We used solvent-based extraction with internal standards added to the sample prior to extraction. The sample and 250–2525 µl of extraction medium so that the ratio sample/extraction medium was kept at 0,002 (chloroform/MeOH/H_2_O; 2∶6∶2) including stable isotope reference compounds were added to an Eppendorf tube. The extraction was performed in an MM 301 vibration mill (Retsch GmbH & Co. KG, Haan, Germany). After extraction, the samples were derivatized 16 h of at room temperature. For the GC-MS analysis, one µL of the derivatized sample was injected by an Agilent 7683 autosampler into an Agilent 6890 gas chromatograph (J&W Scientific). The column effluent was introduced into the ion source of a Pegasus III time-of-flight mass spectrometer, GC-TOFMS (Leco Corp., St Joseph, MI, USA). All data were processed by ChromaTOF (1.00) software (Leco Corp.). Each sample was normalized before the multivariate analysis. Normalization is essential if the samples are not identical, e.g., if there are differences in sample weight or volume, or a purification or derivatization step is involved that might result in variations in recovery. We divided the response values (area counts) by the sample weight and the intensity of one or more internal standards. For a more detailed description of the extraction, derivatization and analysis steps see Gullberg et al. [41].

### Aphid surveys

We performed surveys of aphids from spruce trees with honeydew harvesting ants present, on clear days in July 2006. We used eight sites in each age category. [31]. Aphids were counted and some specimens were identified in the laboratory in 2006. In 2011 we did a more detailed determination of samples from 10 of the stands (4 clear cuts, 3 middle aged stands and 3 old stands) so that the assemblage composition of aphids could be compared among stand types. Three branches were taken from five different spruce trees or saplings in each of the sites. Branches were collected from the upper canopy, just below the tree crown, in all site types as it was not possible to safely reach the crown in old stands. We used ladders to access canopies in middle-aged stands and experienced climbers to reach the canopy of spruce trees in old stands. At all sites, branches were cut at the base and bagged. On the ground, we beat the branches and collected aphids and other insects were from the beating tray using an aspirator. The stands selected for the more detailed analysis were the same stands that were used for the honeydew sampling. However one old stand from the same area but not sampled for honeydew was added to increase the sample size for old stands.

### Mass of honeydew harvested

To compare the mass of honeydew collected by individual ants in different stand ages we collected ants moving up and down trees on fine days in July 2008. Ten spruce trees with *F. aquilonia* activity were selected at each site between 0.5 and 35 m from a central nest. We collected five ants moving up and five ants moving down each of the ten trees at four sites belonging to each of the three stand ages for a total of 1200 ants. We did not specifically select ants with laden and non-laden gasters because we aimed to compare harvesting efficiency per ant. To effectively collect ants we used an aspirator built from a battery driven vacuum cleaner. This method was selected as ants proved less likely to squirt formic acid (and therefore lose weight) when removed using an aspirator than by forceps. Ants were placed in a cold box (approximately 5°C) in the field and were later frozen for 3 days at −20°C to ensure that they were killed. Ants were weighed individually in the lab and any needles or prey items were removed before weighing.

### Statistical analyses

To test for differences in honeydew composition among stand ages we used PERMANOVA (permutational multivariate analysis of variance) in Primer 6 [Primer 42,43]. We performed separate analyses for total honeydew composition, carbohydrates and amino acids. Because the concentrations were not directly comparable among compounds, we standardised each compound (so that the sum of area counts for each compound from all samples was 1 (100%)) and fourth root transformed the data before analysis to both reduce the influence of the most abundant compounds and assess changes in proportional abundance of compounds [44]. We used the Bray-Curtis similarity measure which is not affected by joint absences [45], 5000 permutations of the data, and performed the permutations of residuals under a reduced model. Pair-wise tests were used to detect which stand ages differed from each other [43], [46].

We tested for differences among stand ages for identified carbohydrates and amino acids with relevance to ants using the GLM procedure in SAS 9.1 [47]. After examining the residual plots, the data was log(x+1)-transformed before the analysis to correct for heterogeneity in variances. If significant stand age effects occurred, we examined the effect using Tukey's honestly significant difference (HSD) test.

We tested for differences in abundance of the most abundant aphids and their relation (quota *Cinara pruinosa/Cinara piceicola*) among stand types using Kruskal-Wallis test in JMP 8 [48]. When we found significant differences we performed pair wise comparisons among the stand types using Mann-Whitney U-tests.

We used SMATR (Standardised Major Axis Tests & Routines, Version 2) [49], [50] to test for differences in the relationship between head width and body mass between stands of different ages and ants ascending and descending trees. When slopes were not different, we also tested for differences in the elevation and position of regression lines. We used the step down Bonferroni method of Holm to correct for multiple comparisons within each analysis (ANOVAS for sugars and amino acids, U-tests for aphid abundances and SMATR-analyses) [47], [51].

## Results

### Effects of stand age on honeydew composition

We found 132 compounds in our honeydew samples, including 20 carbohydrates, seven amino acids, nine organic acids, fatty acids, a mineral acid and 85 unidentified compounds. Identified compounds that were not used in our analysis are listed in Table S1. We found no significant differences in the total composition of honeydew or in the composition of amino acids among stands of different ages. For carbohydrates there was a trend of different composition in clear cuts compared with old stands (PERMANOVA, Table 1). However, when the identified sugars and amino acids were analysed separately we found that the concentration of trehalose was smaller in clear-cuts than in middle-aged and mature stands. Similar, non-significant trends could be seen for sucrose, raffinose and melezitose (ANOVA, Table 2). Among amino acids, clear-cuts had the highest concentration of serine, mature stands were intermediate and middle-aged stands had the lowest concentration. A similar, but non-significant trend was detected for glutamine (ANOVA, Table 2). After corrections for multiple comparisons none of the trends for sugars and amino acids were significant. However, the Bonferroni correction procedure is often thought to be overly conservative [52], [53].

**Table 1:** Results from PERMANOVA comparing the chemical composition of honeydew harvested by *Formica aquilonia* among stands of different ages.

Source	Pseudo-F	p
**All compounds**		
Stand age	1.26	0.259
Site(Stand age)	5.05	<0.001
**Carbohydrates**		
Stand age	2.22	0.075
Site(Stand age)	4.80	<0.001
**Amino acids**		
Stand age	1.55	0.227
Site(Stand age)	2.47	0.003

Degrees of freedom are 2 for stand age and 9 for site(stand age).

**Table 2:** F-values, and significance of mixed model ANOVA testing the effect of stand age on honeydew composition and mean ± SE for sugars and amino acids in honeydew harvested by *Formica aquilonia* in stands of different ages.

Compound	ANOVA		CC (K area counts)			MID (K area counts)			OLD (K area counts)		
	Stand age	Site(Stand age)	mean		SE	mean		SE	mean		SE
**SUGARS**											
Erlose	2.45	6.48**	151.6	±	120.4	48.4	±	84.3	4.1	±	8.0
Fructose	0.28	3.16*	1084.2	±	131.1	1099.2	±	172.7	1160.1	±	146.5
Glucose 1	1.34	2.59	2286.9	±	466.6	1865.5	±	448.8	2216.6	±	477.0
Glucose 2	1.8	1	670.7	±	143.3	569	±	150.7	664.7	±	144.1
Lactose	1.86	1.34	6.8	±	10.3	11.3	±	27.3	528.8	±	1750.1
Melezitose	3.13†	4.74**	628.5	±	93.4	842.5	±	139.0	838	±	110.4
Nigerose	1.78	3.78**	2454.9	±	830.3	1692.9	±	943.6	2505.6	±	1140.6
Palatinose	1.62	7.24**	4293.6	±	3567.9	1754.7	±	1621.4	416.3	±	664.6
Raffinose	4.18†	3.43**	1102.2	±	865.0	2277.2	±	1005.5	1438.8	±	485.7
Sucrose	3.85†	6.27**	5.7	±	1.7	9.6	±	2.1	8.3	±	2.1
Trehalose	7.89††	4.30**	2415.2	±	554.9	3762.5	±	707.6	4142.1	±	602.9
Turanose	2.44	4.08**	884.3	±	192.1	649.6	±	265.6	864.7	±	220.6
**AMINO ACIDS**											
Allothreonine/Threonine	1.04	2.32	19.5	±	14.8	7	±	3.9	10.1	±	5.8
Glutamine	3.53†	2.35	96.7	±	205.8	2.1	±	0.7	2.9	±	3.6
Leucine	1.69	2.92*	12.5	±	7.7	5.6	±	3.2	9.7	±	5.2
Ornithine	0.56	1.42	4.3	±	3.5	2.2	±	1.6	3	±	1.7
Phenylalanine	0.24	3.18*	38.1	±	24.6	2.5	±	9.2	26.2	±	10.8
Serine	5.01††	1.23	69.6	±	41.0	26.7	±	14.6	47.5	±	32.3
Valine	0.59	3.89**	9	±	4.3	5.7	±	3.2	4.9	±	2.4

Degrees of freedom are 2 for stand age and 9 for site(stand age).

† = <0.1 before corrections for multiple comparisons;

†† = p<0.05 before corrections for multiple comparisons;

* *p*<0.05;

** *p*<0.01;

Concentrations are presented in kilo area counts as retrieved from the GC/MS analysis. CC = clear cut, MID = middle aged stands, OLD = old stands.

In the aphid survey, we sampled in total 2108 aphids from spruce trees with honeydew harvesting ants present. The samples were dominated by *Cinara piceicola* (1641 specimens) and *Cinara pruinosa* (164 specimens). Four specimens could not be determined and 299 were determined as *Cinara* sp. Both determined *Cinara* species were more abundant in clear cuts compared with middle aged and old stands but the pattern was only significant for *C. piceicola* (Kruskal-Wallis test, p = 0.018). The quota of *C. pruinosa/C. piceicola* did not differ among stand types (Kruskal-Wallis test, p = 0.883) but *C. piceicola* dominated the samples in clear cuts (quota 0.07±0.04, mean±SE) and *C. pruinosa* in old stands (quota 5.33±5.33, mean±SE), middle aged stands were intermediate (quota 0.88±0.82, mean±SE).

### Effects of body size and stand age on forager mass

**Figure 1:**
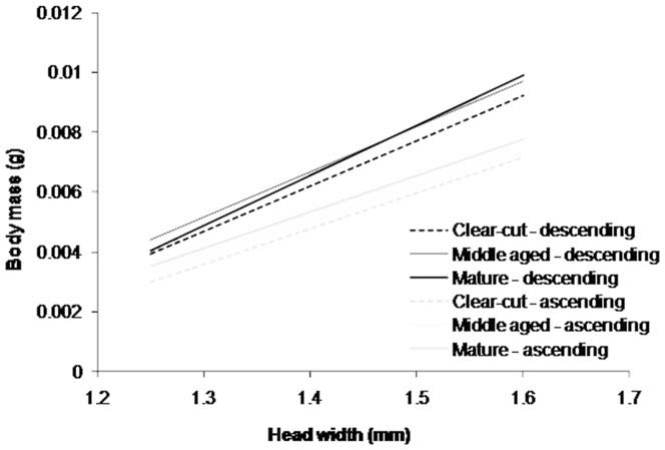
Relationship between body mass and head width for ants ascending and descending trees in stands of different ages. Line equations are: descending ants: clear-cut: y = −0.015+0.015x, R^2^ = 0.65; middle-aged: y = −0.015+0.015x, R^2^ = 0.64; mature: y = −0.017+0.015x, R^2^ = 0.57; ascending ants: clear-cut: y = −0.012+0.012x, R^2^ = 0.73; middle-aged: y = −0.011+0.012x, R^2^ = 0.71; and mature: y = −0.012+0.012x, R^2^ = 0.80.

Ants descending trees were laden and thus heavier than ants ascending trees for all stand ages. The slope of the regression of body mass on head width was significantly lower for ants ascending than ants descending trees for stands of all ages (Table 3, Figure 1). This suggests that ants with larger heads carry more honeydew relative to their mass and may therefore be more efficient. For both ascending and descending ants, there were no differences in slope between stand ages.

**Table 3:** Test-statistic and significance from the SMATR analysis comparing the slopes of lines for the relationship between head width and body mass for *Formica aquilonia* workers ascending and descending trees.

		Clear-cut		Middle-aged		Mature
Stand age	Foragers	Descending	Ascending	Descending	Ascending	Descending
Clear-cut	Ascending	17.2*				
Middle-aged	Descending	0.01	17.4*			
	Ascending	21.2*	0.3	21.4*		
Mature	Descending	2.8	31.2*	2.5	35.7*	
	Ascending	16.8*	0.1	17.1*	0.9	31.3*

* *p*<0.05 after Bonferroni stepwise corrections for multiple comparisons.

We also tested for differences in the elevation of lines with equal slopes, i.e., within the groups of ascending and descending ants. For ascending ants, line elevation differed significantly between stands of different ages (Wald statistic_(2)_ = 24.0, *p*<0.001), with elevation being highest in mature stands (Figure 1). This suggests that ants in mature stands are heavier relative to head width than those in other stand ages. Line position for ascending ants was not affected significantly by stand age (Wald statistic_(2)_ = 4.4, *p* = 0.110).

For descending ants, line elevation differed significantly between stands of different ages (Wald statistic_(2)_ = 10.1, *p* = 0.006) and was significantly lower in clear-cuts than in other stand ages (Figure 1), while line position did not differ between stands of different ages (Wald statistic_(2)_ = 4.0, *p* = 0.134). This suggests that ants on clear-cuts may harvest slightly less honeydew in proportion to their head width.

## Discussion

### Honeydew composition

Both sugar and amino acid concentrations tended to differ between stands of different ages, indicating that the quality of honeydew changes in response to forest management. Trends were similar for both univariate and multivariate analyses. The concentration of several sugars was lowest in clear-cuts. This trend was significant only for the disaccharide trehalose and only before corrections for multiple comparisons. Trehalose is the blood sugar of insects and typically makes up 30–35 percent of the honeydew sugar content [20], but is also found in the haemolymph of ants [54]. The origin of the trehalose in our samples is most probably honeydew because cloudy samples representing contamination with haemolymph were not used in the analysis. The nutritional value of trehalose relative to other sugars is unclear, but lower concentrations of trehalose and other sugars in honeydew on clear-cuts may contribute to the reduced success of *F. aquilonia*.

Raffinose, sucrose, and melezitose showed a similar trend to trehalose. Sucrose is nutritionally important for ants [55], [56], but the value of melezitose, which commonly makes up 50–60 percent of honeydew sugars, is unclear [20], [57]. Mutualist aphids increase the melezitose content of honeydew when tended [58], possibly because melezitose indicates the presence of sugar rich honeydew to ants (e.g. *Lasius niger* on *Tanacetum vulgare*, Germany) [22]. Although melezitose has been reported to be of nutritional value to some ants [59], other studies suggest that it is of low nutritional value to most insects [60] and can even be toxic [61], so may be merely tolerated by ants [56]. The sugar content of honeydew in combination with the presence of melezitose may thus be the critical factor in determining the extent of ant-attendance in aphids [62]. If melezitose is produced in response to ant tending, lower concentrations of melezitose on clear-cuts may indicate that aphids are attended less frequently by ants than in other stand ages. Ant activity on trees on clear-cuts is low relative to mature forests, yet aphids are extremely abundant on saplings in clear-cuts [31], suggesting that aphids may indeed be tended less in these sites.

In addition to sugars frequently observed in honeydew from aphids, our chemical analyses with GC-TOFMS identified several additional sugars e.g. nigerose and palatinose (Table 2), but they did not differ in concentrations among stand types. As we don't know the absolute concentrations of these sugars it is hard to estimate their relative importance. Further studies with this improved technology (GC-TOFMS) including measures of absolute concentrations are needed to reveal the function and relative importance of more unusual sugars in honeydew.

Concentrations of several amino acids were higher on clear-cuts than in older forests. Clear-cutting leads to increased availability of nitrogen in the soil for several years after cutting [63], [64] and soil scarification further speeds up this effect [65], [66]. Trees on clear cuts also contain more N in the needles than trees in older stands [67], so increased N-uptake by the trees after clear cutting may explain the increased amino acid content of aphid honeydew. Of the amino acids affected, serine is a major precursor to formic acid [68], so may be critical in colony defence behaviours, while glutamine is a storage protein for workers and reproductive castes [69]. Ant species that commonly collect nectar or honeydew may exhibit higher preferences for sources rich in proteins or amino acids [70]. This may reflect nitrogen limitation in their diet [71], which could be a result of decreases in prey availability after clear-cutting [72]. It is thus possible that increased nitrogen availability in honeydew on clear-cuts has positive effects on some aspects of ant biology.

The aphid surveys revealed that the ant tended *Cinara pruinosa* and *Cinara piceicola* dominated as honeydew producers in all stand types so it is highly probable that the collected honeydew arise from these two species. The numbers of aphids varied both among and within stands and both species often occurred on the same tree so it was impossible to know which species was the source of the honeydew. The higher abundance of both species on clear cuts might be a result of the sampling procedure since it was not possible to reach the tree crowns in middle aged and old stands. Although *Cinara piceicola* dominated the samples in clear cuts and *C. pruinosa* in old stands with middle aged stands intermediate the statistical analyses did not reveal a difference in the quota *C. pruinosa/C. piceicola*, probably because the low sample size in old stands. Honeydew composition differs among aphid species but also within species e.g. depending on host plant and ant attendance [58]. Because the same species of ant-attended aphids dominated in all stand ages in this study, and honeydew was collected from ants that highly probably had harvested their honeydew from aphid-colonies tended by the ants, differences in honeydew composition are likely to depend on site factors rather than differences in the source of honeydew.

### Determinants of honeydew harvest by individual workers

Head width-mass slopes were steeper for descending than ascending ants, indicating that large ants harvested more honeydew than small ants. Ascending ants in mature forests weighed more for their size, suggesting that they may have been in better condition than ants in younger stands. However, they did not harvest more honeydew than ants in other stand ages. Both ascending and descending ants in clear-cuts weighed less than those in middle-aged and mature forests. Consistent with our findings, Sorvari and Hakkarainen (2009) showed that *Formica aquilonia* worker size, body fat-content and worker-generated nest temperature all decrease in response to clear-cutting. Previous studies suggest that food availability may determine the size of ant workers [24], [73] and that large workers may be superior foragers and defenders of nests [26], [74]. However, differences in the mass gain of ants between stands of different ages at these study sites were small and non-significant [31], even with a large sample size, so consequences for the colony are difficult to determine.

Despite suggestions that honeydew limitation is responsible for the decline of wood ants after clear-cutting [25], [29], [75], results from a previous study in the same stands show that in clear-cuts, ants harvest 77% of the honeydew mass that they harvest in mature stands [31]. This is probably a result of the high abundance of seedlings with high aphid loads in clear-cuts, which may compensate for the lack of mature trees. We thus suggest that changes in honeydew composition after clear-cutting may also play an important role in the inferior worker condition on clear-cuts.

### Implications for conservation

This is the first study to show that honeydew quality is affected by anthropogenic disturbances. It suggests that changes in resource quality resulting from clear-cutting cascade through food chains. This may be particularly important for closely interacting species, such as those involved in mutualisms. We suggest that the reported poorer condition of *F. aquilonia* on clear-cuts is the result of changes in honeydew quality. Changes in food quality may thus exacerbate the changes in resource quantity and microclimate reported in previous studies [31], [35] and thus explain reported reductions in size and abundance of *Formica aquilonia* mounds following clear-cutting. Other honeydew-dependent organisms, such as microbes, may also be affected by this change in quality [76], [77]. Thus, changes in honeydew quality due to forestry may have further consequences for species and ecosystem functions beyond those reported here.

## Supporting Information

Table S1 Mean and SE for chemical compounds in honeydew harvested by *Formica aquilonia* in different stand ages. Concentrations are presented in kilo area counts as retrieved from the GC/MS analysis (K area counts). Data for the sugars and amino acids that were used in the statistical analyses are shown in Table 2.
(DOC)
