# Supplementary material for: Forestry Alters Foraging Efficiency and Crop Contents of Aphid-Tending Red Wood Ants, Formica aquilonia
Source: PLoS One. 2012 Mar 13;7(3):e32817. doi: 10.1371/journal.pone.0032817 (PMC3302777; doi:10.1371/journal.pone.0032817)
Supplement: Table S1 — Mean and SE for chemical compounds in honeydew harvested by Formica aquilonia in different stand ages. Concentrations are presented in kilo area counts as retrieved from the GC/MS analysis (K area counts). Data for the sugars and amino acids that were used in the statistical analyses are shown in Table 2. (DOC) [file pone.0032817.s001.doc]

**Appendix**. Mean and SE for chemical compounds in honeydew harvested by *Formica aquilonia* in different stand ages. Concentrations are presented in kilo area counts as retrieved from the GC/MS analysis (K area counts). Data for the sugars and amino acids that were used in the statistical analyses are shown in Table 2.

|  | **Clear-cut** |  |  |  | **Middle-aged** |  |  |  | **Mature** |  |  |
| --- | --- | --- | --- | --- | --- | --- | --- | --- | --- | --- | --- |
|  | **Mean** |  | **SE** |  | **Mean** |  | **SE** |  | **Mean** |  | **SE** |
| **Aminoalcohol** |  |  |  |  |  |  |  |  |  |  |  |
| Ethanolamine like | 598.43 | ± | 136.38 |  | 548.45 | ± | 111.53 |  | 648.29 | ± | 217.99 |
| **Carbohydrates** |  |  |  |  |  |  |  |  |  |  |  |
| D-Pinitol | 2.17 | ± | 0.80 |  | 2.36 | ± | 0.61 |  | 2.50 | ± | 0.66 |
| Galactinol | 221.35 | ± | 135.74 |  | 124.99 | ± | 53.04 |  | 147.09 | ± | 79.75 |
| Glycerol | 773.09 | ± | 355.27 |  | 716.45 | ± | 212.28 |  | 755.31 | ± | 210.58 |
| Inositol | 426.87 | ± | 168.19 |  | 302.79 | ± | 126.32 |  | 216.54 | ± | 68.07 |
| **Fatty acids** |  |  |  |  |  |  |  |  |  |  |  |
| Hexadecanoic acid | 8.94 | ± | 2.64 |  | 7.75 | ± | 1.58 |  | 9.31 | ± | 3.06 |
| Stearic acid | 87.49 | ± | 27.55 |  | 76.72 | ± | 20.75 |  | 98.96 | ± | 49.15 |
| **Hydrocarbon** |  |  |  |  |  |  |  |  |  |  |  |
| Dodecane | 18.77 | ± | 4.88 |  | 17.00 | ± | 4.65 |  | 19.85 | ± | 7.35 |
| **Lipids** |  |  |  |  |  |  |  |  |  |  |  |
| Digalactosylglycerol | 64.70 | ± | 36.38 |  | 89.92 | ± | 31.35 |  | 73.51 | ± | 20.35 |
| Galactosylglycerol | 95.58 | ± | 59.55 |  | 37.78 | ± | 16.66 |  | 55.49 | ± | 20.57 |
| Mineral acid |  |  |  |  |  |  |  |  |  |  |  |
| Phosphoric acid | 254.40 | ± | 109.22 |  | 313.31 | ± | 73.35 |  | 237.08 | ± | 75.42 |
| **Organic acids** |  |  |  |  |  |  |  |  |  |  |  |
| Citric acid | 492.11 | ± | 158.57 |  | 665.78 | ± | 207.27 |  | 933.95 | ± | 285.41 |
| Dehydroabietic acid-Like | 1.41 | ± | 2.19 |  | 2.97 | ± | 4.64 |  | 1.98 | ± | 5.64 |
| Dehydroascorbic acid dimer | 103.01 | ± | 36.75 |  | 81.70 | ± | 31.48 |  | 96.41 | ± | 37.28 |
| Fumaric acid | 61.60 | ± | 48.11 |  | 20.51 | ± | 9.73 |  | 10.46 | ± | 3.98 |
| Gluconic acid | 157.69 | ± | 266.22 |  | 16.45 | ± | 26.09 |  | 14.54 | ± | 15.44 |
| Malic acid | 199.78 | ± | 135.88 |  | 369.77 | ± | 481.19 |  | 279.47 | ± | 136.51 |
| Quinic acid | 2.60 | ± | 0.94 |  | 2.11 | ± | 0.64 |  | 2.59 | ± | 0.86 |
| Shikimic acid | 343.78 | ± | 92.96 |  | 717.62 | ± | 202.04 |  | 787.59 | ± | 186.50 |
| Succinic acid | 16.41 | ± | 3.34 |  | 13.66 | ± | 2.53 |  | 11.92 | ± | 2.02 |
| **Other** |  |  |  |  |  |  |  |  |  |  |  |
| Salicylic acid-glucopyranoside | 27.51 | ± | 14.76 |  | 20.41 | ± | 7.32 |  | 25.60 | ± | 12.88 |
| **Sugar alcohol** |  |  |  |  |  |  |  |  |  |  |  |
| Mannitol | 6303.26 | ± | 2287.45 |  | 9535.44 | ± | 2817.04 |  | 11116.46 | ± | 4115.65 |
